# Prevalence and antimicrobial resistance profiles of *Salmonella* species and *Escherichia coli* isolates from poultry feeds in Ruiru Sub-County, Kenya

**DOI:** 10.1186/s13104-021-05456-4

**Published:** 2021-02-02

**Authors:** Dorica Gakii Ngai, Anthony Kebira Nyamache, Omwoyo Ombori

**Affiliations:** 1 Department of Microbiology, Kenyatta University, P.O Box 43844-00100, Nairobi, Kenya; 2 Department of Pant and Microbial Sciences, Kenyatta University, P.O Box 43844-00100, Nairobi, Kenya

**Keywords:** Poultry feed contamination, *Salmonella*, *E. coli*, Antimicrobial resistance, Resistance genes

## Abstract

**Objectives:**

Contaminated poultry feeds can be a major source of *E. coli* and *Salmonella* infections in poultry. This study aimed at determining microbial load, prevalence and antimicrobial resistance profiles of *Salmonella* sp. and *E. coli* and associated resistance genes among isolates from poultry feeds.

**Results:**

A total of 150 samples of different poultry feed types were randomly collected from selected sites within Ruiru Sub-County. The microbial load was determined, *Salmonella* sp. and *Escherichia coli* were isolated and antimicrobial susceptibility test carried out. Antimicrobial resistance genes were also screened among the resistant isolates. Out of analyzed samples, 58% and 28% contained *Escherichia coli* and *Salmonella* sp. respectively. Bacterial load ranged between 3.1 × 10^5^ and 3.0 × 10^6^ cfu/g. Highest resistance was against ampicillin (41%) for *Salmonella* sp. and (62%) for *E. coli* isolates. Ampicillin resistant isolates carried *TEM* and *SHV* genes. In addition, *strB* and *Dfr* resistance genes associated with streptomycin and cotri-moxazole were detected. All the isolates were susceptible to chloramphenicol and ciprofloxacin. The study reveals high bacterial contamination, presence of beta-lactamase, aminoglycoside and sulphonamide resistance genes across isolates from poultry feeds. Therefore, contaminated poultry feeds with bacteria are likely to lead to increase in antimicrobial resistant strains across the community.

## Introduction

Poultry feeds are contaminated with microbes during harvesting, preparation and sale of the produced feeds [1]. Poultry feed contamination has been associated with *Escherichia coli* and *Salmonella* species [2]. Antimicrobial resistance is a concern with medical and veterinary sciences due to its effects on public health [3]. Indiscriminate use of antimicrobials may increase antibiotic resistance in nonpathogenic and pathogenic bacteria [4].

Antibiotics, enzymes, pigments and antifungals are non-nutritive additives in feed formulations that help in maintaining health status of the poultry [5, 6]. The intensive use of antibiotics in poultry production [7] has led to antibiotic resistance in almost all common class of antibiotics [8]. Antibiotics in poultry promote growth, treat, control, and prevent infectious diseases [5, 9, 10]. However, the beneficial uses of antibiotics in poultry have been affected by emergence of resistant strains of bacteria [11]. Different studies have been carried out to determine the existence of *Salmonella* and *E*. *coli* in domestic fowl feeds and antibiotic resistance patterns on isolates from poultry feed [12–14]. Nonetheless, measures to control use of antibiotics in food animals and investigations on associated resistant genes in poultry feed are minimal in developing countries.

In Kenya, increased poultry rearing has increased the demand for feeds leading to more feed companies. Microbiological health controls during preparation of poultry feeds are important to minimize contamination of poultry feeds [15]. However, there are minimal reports on bacterial load, profiles of antimicrobial resistance, associated resistance genes and potential source of *Salmonella* and *E. coli* contamination in poultry feeds in Kenya.

This research determined the bacterial load, prevalence of *Salmonella* sp. and *Escherichia coli*, the antimicrobial resistance patterns and assessed presence of resistance genes in poultry feed in Ruiru Sub-County Kenya.

## Main text

### Methods

#### Sampling

A cross-sectional study was carried out between January and April 2019. A total of 150 poultry feed samples was picked up randomly from selected outlets in Biashara, Gitothua, Gatong’ora, Kiuu and Mwihoko in Ruiru Sub County, Kenya. Different types of poultry feed that had been produced recently were selected; these included grower mash, layer mash, starter mash, finisher mash, kienyeji mash, chick mash, maize germ and sunflower. Estimated 400 g of each sample was collected aseptically in collection bags and taken to Kenyatta University Microbiology laboratory for analysis.

Before commencement of this study, permission was sort and granted from Commissions for Science Technology and Innovation and County Commissioner, Kiambu County.

#### Bacteriological analysis

One gram of feed sample was homogenized in 9 ml sterile deionized water, thoroughly mixed to form a ratio of 1:10 and a fourfold serial dilutions were made. Aliquot of 0.1 ml of the serial dilution was drawn and inoculated into nutrient agar (Oxoid) using the spread plate method. The inoculants were then incubated at 37 °C for 18–24 h. Colonies were counted using colony counter and total bacterial count calculated; CFU/g = level of Dilution plated x number of colonies counted/ amount plated [16]. Bacterial counts below 30 and above 300 were excluded since they were not within the statistically proven range of colonies to be considered when determining total count of bacteria [17].

#### Isolation and identification of *Salmonella* sp. and *Escherichia coli*

Samples were enriched in selenite F broth and peptone water (Oxoid) and incubated at 37 °C for 18 h. Thereafter, Samples enriched in selenite F broth were inoculated onto Xylose Lysine Deoxycholate (XLD) agar and Salmonella-Shigella (SS) agar (Oxoid) for selection of *Salmonella* sp. Samples enriched in peptone water were inoculated onto sorbitol MacConkey agar (Oxoid). The inoculated cultures were then incubated at 37 °C for 24 h and biochemical tests employed to confirm suspected *Salmonella* sp. and *Escherichia coli* as previously described [18, 19].

#### Antimicrobial susceptibility test

Kirby–Bauer disc diffusion technique was applied to establish the susceptibility of isolates to antibiotics [20]. Antimicrobial agents; ampicillin (10 µg), ceftriaxone (30 µg), co-trimoxazole (25 µg), tetracycline (30 µg), chloramphenicol (50 µg), ciprofloxacin (30 µg) (Oxoid) were evaluated using *Escherichia coli* ATCC 25,922 as control organism. Antimicrobial susceptibility results were elucidated according to Clinical and Laboratory Standard Institute guidelines [21].

#### Extraction of bacteria genomic DNA

Bacteria DNA was extracted using boiling method as earlier described [22, 23]. One milliliter of overnight bacterial culture of the pooled 34 bacterial resistant isolates were suspended in 1000 μl of sterile distilled water and then boiled for 18 min at 100 °C. The resulting suspension was then centrifuged for 5 min at 14,200 rpm to sediment the debris and the supernatant was stored at − 20 °C for subsequent use as the DNA template.

#### Amplifications of drug associated resistance genes

Resistance genes encoding resistance to betalactams; *SHV* (sulphydryl variable enzyme), *TEM* (temoneira), sulpnomide; *Dfr* (dihydroflavonol 4-reductase enzyme), aminoglycoside; *strB* (streptomycin) genes were assessed as previously described [19, 20]. *Escherichia coli* ATCC 25922 known to carry different antimicrobial resistance genes was used as the positive control. Polymerase Chain Reaction amplification products were validated by visualization using gel electrophoresis as previously described [24].

#### Data analysis

The prevalence was calculated as positive samples as a percentage of total samples collected. Data on antimicrobial resistance was interpreted as resistant, intermediate or susceptible. Analysis of variance was used to determine the variability of bacterial loads among the samples and Tukey’s HSD at a significance of 0.05 was used to separate the means.

### Results

#### Microbial load in poultry feeds

A total of 150 samples were analyzed including layer mash, grower mash, chick mash, kienyeji mash, starter mash, finisher mash and maize germ/sunflower. Thirty seven samples were excluded since their colony count was not within the required range of 30–300 colonies. Bacterial load ranged from 3.1 × 10^5^ to 3.0 × 10^6^ cfu/g. The highest bacterial load was detected in layer mash (Table 1). There was a significant difference in bacterial load among the poultry feeds (p = 0.0001).

**Table 1:** Range of bacterial load in poultry feeds

Feed type	Number of samples	Number of samples excluded	Bacterial load (cfu/g)	
			Lowest	Highest
Layer mash	29	5	3.2 × 10^5^	3.0 × 10^6^
Grower mash	27	7	3.5 × 10^5^	2.04 × 10^6^
Chick mash	30	2	3.2 × 10^5^	2.55 × 10^6^
Kienyeji mash	27	5	3.1 × 10^5^	2.9 × 10^6^
Starter mash	27	4	3.2 × 10^5^	2.94 × 10^6^
Finisher mash	6	2	7.0 × 10^5^	1.37 × 10^6^
Maize germ/sunflower	4	2	4.2 × 10^5^	1.01 × 10^6^
Total	150	37		

#### Prevalence of *Salmonella* sp. and *E. coli*

Out of the 150 samples, 58% were detected with *Escherichia coli* and 28% with *Salmonella* sp. The prevalence of *Salmonella* sp. in different poultry feeds ranged from 17 to 38% while the prevalence of *Escherichia coli* was between 33 and 100% among the different types of poultry feeds (Table 2).

**Table 2:** Prevalence of *Salmonella* sp. and *Escherichia coli* in different poultry feed

Feed type	Total samples	Positive samples	Positive samples
		*Salmonella* sp.	*Escherichia coli*
Layers mash	29	11 (38%)	18 (62%)
Growers mash	27	10 (37%)	14 (52%)
Starter mash	27	5 (19%)	13 (48%)
Finisher mash	6	1 (17%)	2 (33%)
Kienyeji mash	27	5 (19%)	17 (63%)
Chick mash	30	9 (30%)	19 (63%)
Others	4	1 (25%)	4 (100%)
Total	150	42 (28%)	87 (58%)

#### Antimicrobial susceptibility profiles for *Salmonella* sp. and *Escherichia coli*

The *Salmonella* sp. showed diverse response to different antibiotics used. All *Salmonella* sp. were susceptible to ciprofloxacin, chloramphenicol and streptomycin. However, among the tested isolates, 41% were resistant to ampicillin, 2% to co-trimoxazole, 5% to ceftriaxone and tetracycline. Intermediate ranged between 0 and 19% (Table 3).

**Table 3:** Antimicrobial susceptibility profiles of *Salmonella* sp.

Antibiotic class	Antibiotic	Susceptible	Intermediate	Resistance
		n (%)	n (%)	n (%)
Betalactam	Ampicillin	17 (41%)	8 (19%)	17 (41%)
	Ceftriazone	37 (88%)	3 (7%)	2 (5%)
Sulphonamide	Co-trimoxazole	41 (98%)	0%	1 (2%)
Tetracycline	Tetracycline	39 (93%)	1 (2%)	2 (5%)
Phenicols	Chrolamphenicol	42 (100%)	0%	0%
Fluoroquinolones	Ciprofloxacin	35 (83%)	7 (17%)	0%
Aminoglycosides	Streptomycin	41 (98%)	1 (2%)	0%

*Escherichia coli* isolates showed highest resistance against ampicillin (71%). Resistance to other antibiotics ranged from 1 to 10% with no isolate showing resistance to ciprofloxacin. *Escherichia coli* isolates were 100% susceptible to ciprofloxacin, followed by chloramphenicol (98%), co-trimoxazole (89%), tetracycline and streptomycin (86%) and ceftriaxone (78%). The highest intermediate was observed in ampicillin with 21% (Additional file 1: Table S1).

#### Antimicrobial resistance genes among the isolates

The total number of isolates screened for resistance genes was 34. Among the screened isolates, *TEM*, *SHV*, *Dfr* and *strB* genes were detected (Additional file 2: Figure S1, Additional file 3: Figure S2, Additional file 4: Figure S3, Additional file 5: Figure S4). The *TEM* gene dominated with 24%, followed by *Dfr* 21%, *SHV* 12% and *strB* 9% (Additional file 6: Table S2).

### Discussion

The recorded prevalence of 28% of *Salmonella* sp. in poultry feeds was similar to previous prevalence of 29% of *Salmonella* isolates reported in Tanzania [25] and Bangladesh [26]. Nevertheless, studies in Africa have continued to show varying results on prevalence of *Salmonella* in poultry feeds. A prevalence of 38% and 31% was reported in Nigeria [27, 28] and a prevalence of 71%, 55% and 29% in Bangladesh [15, 19, 26]. The prevalence of *Salmonella* sp. from different poultry feeds was 38%, 37%, 19% and 17% in layer mash, grower mash, starter mash and finisher mash, respectively, contrary to previous studies that recorded prevalence of 20%, 0%, 40% and 25% in layer mash, grower mash, starter mash and finisher mash, respectively [14] and prevalence of 21%, 38%, 31% and 33% in layer mash, grower mash, starter mash and finisher mash respectively in Tanzania [25]. The disparities of *Salmonella* prevalence could be due to differences in sampling, testing methods and difficulties in *Salmonella* detection methods [25].

The prevalence of 58% of *Escherichia coli* obtained was similar to a related study carried out in Bangladesh that reported prevalence of 57% of *Escherichia coli* [26]. Other related studies reported different prevalence of 16% in Iraq [1] and in Nigeria 10.6% and 11% [27, 29]. Prevalence of bacteria varies considerably depending on nature of production, country and detection methods applied [30]. Microbial contamination of poultry feeds of plant and animal origin has also been associated with harvesting, manufacturing and climatic conditions encountered [12]. Therefore, it is important to reinforce hygienic handling of feeds and preventive control measures to minimize the danger of potential animal and human health hazards.

The different types of poultry feeds recorded varied bacterial load. The varying results in this study could be attributed to methods of harvesting raw materials, different climatic conditions, food formulation, and storage and transportation technologies [12, 27]. The high bacteria count in layer mash could be due to use of fish wastes as animal proteins which harbors heavier bacterial growth [14]. The differences in bacterial load in different types of poultry feed could also be as a result of mixed infections with other microbes and different management practices [31]. Findings in this study indicates that contaminated poultry feed could be source of infections and thus not fit for animal consumption [32, 33].

The current study recorded different antimicrobial resistance patterns of *Salmonella* sp. and *E. coli*. Both isolates of *Salmonella* and *E. coli* registered the highest resistance to ampicillin, 41% and 71% respectively, and highest susceptibility to ciprofloxacin, 83% and 100% respectively. The isolates of *E. coli* indicated highest resistance to ampicillin 71%, followed by tetracycline 10%, co-trimozaxole and ceftriazone 7% and ciprofloxacin 0%. This was similar to a previous study in Bangladesh that recorded ciprofloxacin as the most effective antibiotic against *E. coli* isolates from poultry feeds [34]. Contrary to previous related study in Bangladesh that reported resistance of 30% to ciprofloxacin, 20% to gentamicin, 60% to nalidixic acid and 0% to ceftriaxone among *Salmonella* isolates from poultry feeds, the current study recorded the highest resistance to ampicillin 41%, tetracycline and ceftriaxone 5%, co-trimoxazole 2% and ciprofloxacin 0% [35]. In Kenya, a study on antimicrobial resistance in *Salmonella* and *E. coli* isolates from poultry wastes reported high resistance to amoxicillin which is a beta-lactam, followed by tetracycline and co-trimoxazole [36]. High resistance to co-trimoxazole, beta-lactams and tetracycline among bacterial isolates from chicken in Kenya was also reported [37, 38]. This suggests possible transmission of antibiotic resistant bacteria through poultry feed to poultry.

The resistant isolates of *Salmonella* sp. and *Escherichia coli* carried *TEM* and *SHV* genes. *Dfr* and *strB* genes were also carried among the resistant isolates of *Salmonella* sp*.* and *Escherichia coli*. Most of studies on poultry feeds have not reported on antimicrobial resistance genes [26, 35, 39]. Nevertheless, a study in India on antimicrobial resistance reported absence of major extended spectrum beta-lactamase in *Salmonella* isolates from poultry feeds [40].

Therefore, poultry feeds are potential source of antimicrobial resistant genes that can be transferred to poultry and humans and pose a health threat to the society.

### Conclusion

This study found out that poultry feeds harbor bacteria and resistance genes. This indicated a threat to public health including humans and animals. It’s important for poultry feeds to be assessed for microbial quality by manufacturers and health authorities to facilitate feed safety.

## Limitation

This study never assessed the source of the resistant genes. The acquisition of the resistance genes could be from humans during processing of the feeds or prior contamination of raw materials of the feeds with microorganisms.

## Supplementary Information

**Additional file 1: Table S1.** Antimicrobial susceptibility profiles of *Escherichia coli*.

**Additional file 2: Figure S1.** PCR amplification of *TEM* genes.

**Additional file 3: Figure S2.** PCR amplification of *SHV* genes.

**Additional file 4: Figure S3.** PCR amplification of *strB* genes.

**Additional file 5: Figure S4.** PCR amplification of *Df*r genes.

**Additional file 6: Table S2.** Distribution of resistance genes.

## Data Availability

The data sets used and/or analysed during the current study are available from the corresponding author on reasonable request.

## Abbreviations

*SHV*: Sulphydryl variable enzyme
*Dfr*: Dihydrofolate reductase
*TEM*: Temoneira
*strB*: Streptomycin resistant gene
