# Supplementary material for: Prevalence and antimicrobial resistance profiles of Salmonella species and Escherichia coli isolates from poultry feeds in Ruiru Sub-County, Kenya
Source: BMC Res Notes. 2021 Feb 2;14:41. doi: 10.1186/s13104-021-05456-4 (PMC7852182; doi:10.1186/s13104-021-05456-4)
Supplement: Supplementary file 1 — Additional file 1: Table S1. Antimicrobial susceptibility profiles of Escherichia coli. [file 13104_2021_5456_MOESM1_ESM.docx]

**Table S1:** Antimicrobial susceptibility profiles of *Escherichia coli*

| **Antibiotic class** | **Antibiotic** | **Susceptible** | **Intermediate** | **Resistance** |
| --- | --- | --- | --- | --- |
|  |  | **n (%)** | **n (%)** | **n (%)** |
| **Betalactam** | Ampicillin | 7 (8 %) | 18 (21 %) | 62 (71 %) |
|  | ceftriaxone | 68 (78 %) | 13 (15 %) | 6 (7 %) |
| **Sulphonamide** | Co-trimoxazole | 77 (89 %) | 4 (5 %) | 6 (7 %) |
| **Tetracycline** | Tetracycline | 75 (86 %) | 3 (3 %) | 9 (10 %) |
| **Phenicols** | Chrolamphenicol | 85 (98 %) | 1 (1 %) | 1 (1 %) |
| **Fluoroquinolones** | Ciprofloxacin | 87 (100 %) | 0 % | 0 % |
| **Aminoglycosides** | Streptomycin | 75 (86 %) | 10 (11 %) | 2 (2 %) |
