# Supplementary material for: Prevalence and antimicrobial resistance profiles of Salmonella species and Escherichia coli isolates from poultry feeds in Ruiru Sub-County, Kenya
Source: BMC Res Notes. 2021 Feb 2;14:41. doi: 10.1186/s13104-021-05456-4 (PMC7852182; doi:10.1186/s13104-021-05456-4)
Supplement: Supplementary file 2 — Additional file 2: Figure S1. PCR amplification of TEM genes. [file 13104_2021_5456_MOESM2_ESM.docx]

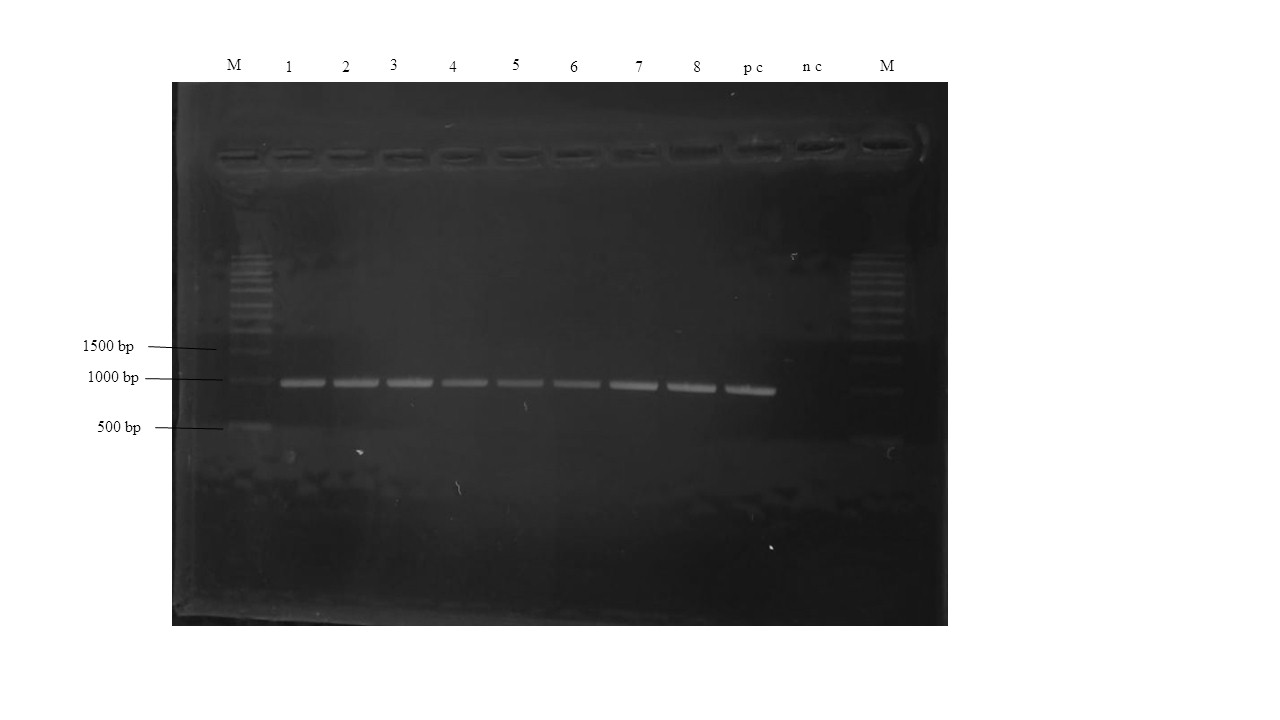


Figure S1: PCR amplification of 980bp size *TEM* gene fragment

Key: M:1 kb DNA ladder, 1-4 *Salmonella* isolates from starter mash, starter mash, layers mash, chick mash respectively, 5-8 *E. coli* isolates from kienyeji mash, layers mash, chick mash and starter mash respectively. p c: positive control, n c: negative control.
