# Supplementary material for: Prevalence and antimicrobial resistance profiles of Salmonella species and Escherichia coli isolates from poultry feeds in Ruiru Sub-County, Kenya
Source: BMC Res Notes. 2021 Feb 2;14:41. doi: 10.1186/s13104-021-05456-4 (PMC7852182; doi:10.1186/s13104-021-05456-4)
Supplement: Supplementary file 3 — Additional file 3: Figure S2. PCR amplification of SHV genes. [file 13104_2021_5456_MOESM3_ESM.docx]

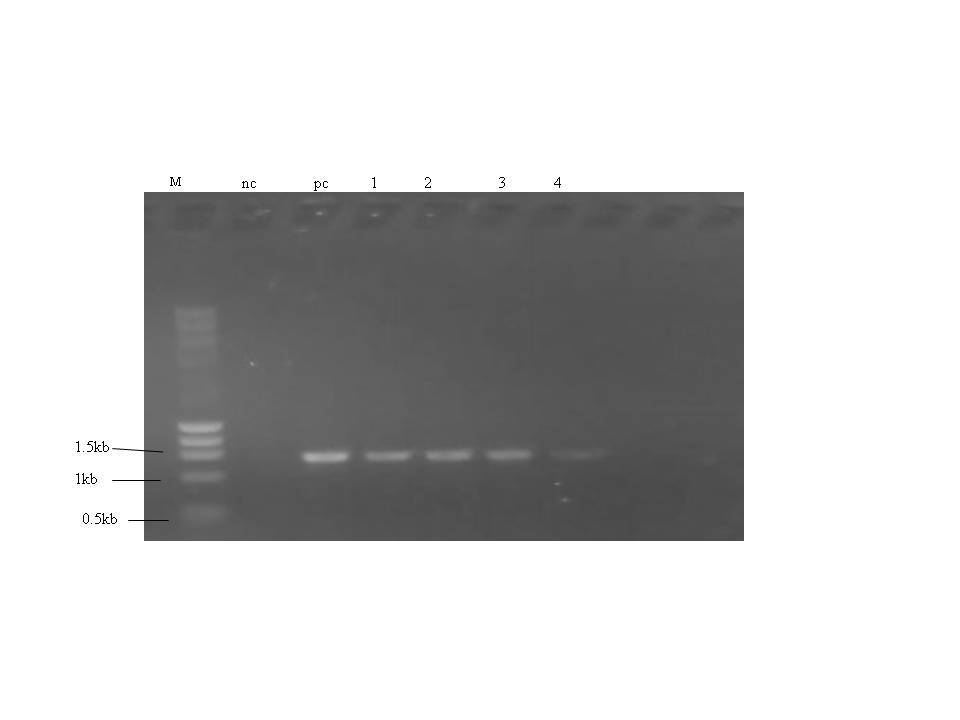


Figure S2: PCR amplification of 1200bp size *SHV* gene fragment

Key: M:1kb DNA ladder, 1 *Salmonella* isolates from growers mash, 2-4 *E. coli* isolates from kienyeji mash, growers mash, chick mash respectively, pc- positive control, n c-negative control
