# Supplementary material for: Prevalence and antimicrobial resistance profiles of Salmonella species and Escherichia coli isolates from poultry feeds in Ruiru Sub-County, Kenya
Source: BMC Res Notes. 2021 Feb 2;14:41. doi: 10.1186/s13104-021-05456-4 (PMC7852182; doi:10.1186/s13104-021-05456-4)
Supplement: Supplementary file 4 — Additional file 4: Figure S3. PCR amplification of strB genes. [file 13104_2021_5456_MOESM4_ESM.docx]

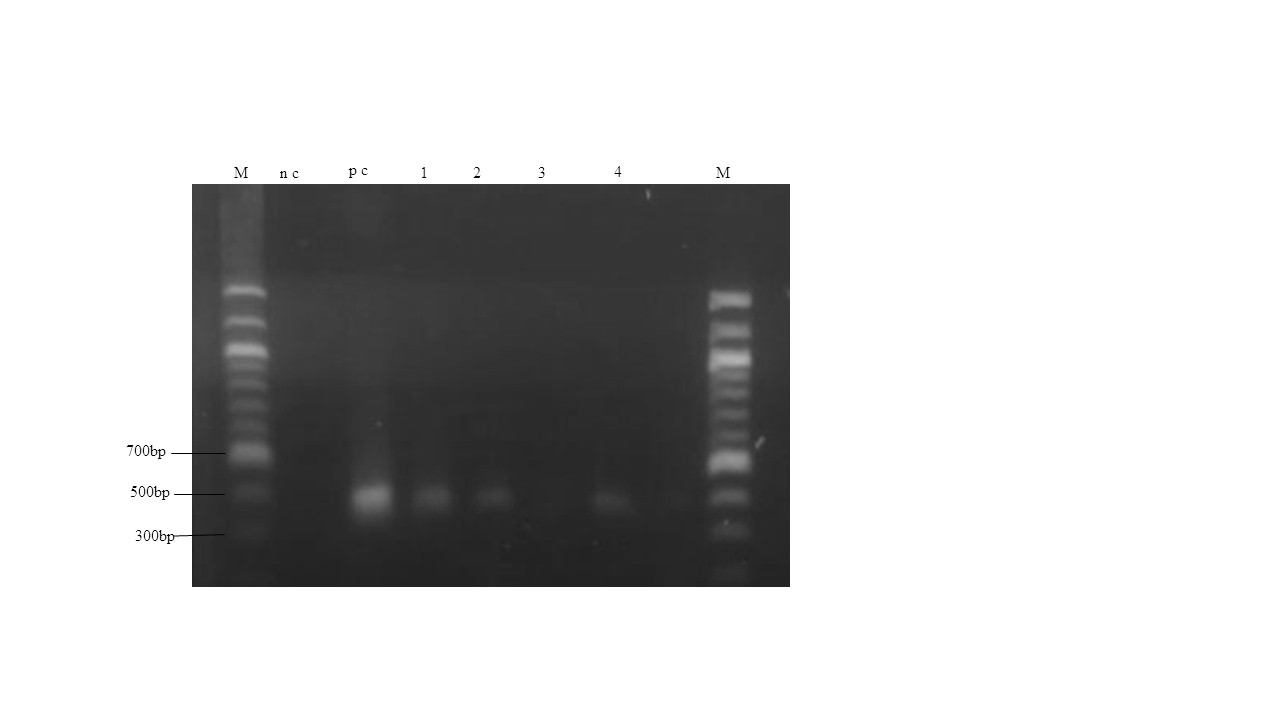


Figure S3: PCR amplification of 400bp size *strb* gene fragment

Key: M: 1 kb DNA ladder, 1-4 *E. coli* isolates from kienyeji mash, chick mash, kienyeji mash, growers mash respectively; nc-negative control, pc positive control .
