# Supplementary material for: Prevalence and antimicrobial resistance profiles of Salmonella species and Escherichia coli isolates from poultry feeds in Ruiru Sub-County, Kenya
Source: BMC Res Notes. 2021 Feb 2;14:41. doi: 10.1186/s13104-021-05456-4 (PMC7852182; doi:10.1186/s13104-021-05456-4)
Supplement: Supplementary file 5 — Additional file 5: Figure S4. PCR amplification of Dfr genes. [file 13104_2021_5456_MOESM5_ESM.docx]

**
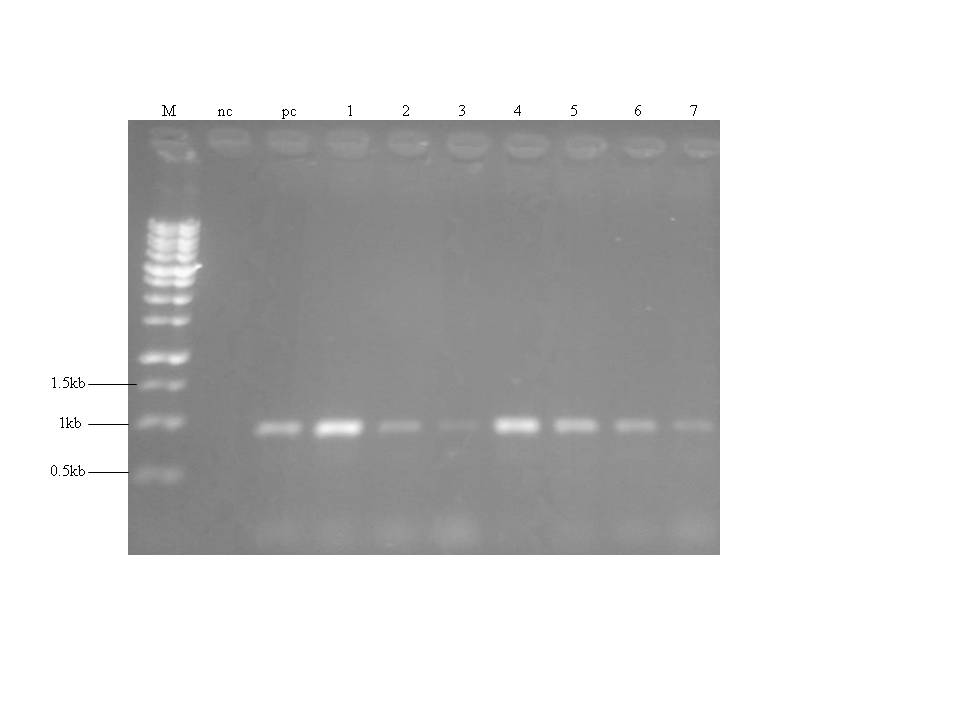
**

Figure S4: PCR amplification of 800bp size *dfr* gene fragment

Key: M: 1 kb DNA ladder, 1 *Salmonella* isolate from growers mash, 2-7 *E. coli* isolates from growers mash, growers mash, kienyeji mash, layers mash starter mash respectively, n c-negative control, p c positive control
