# Supplementary material for: Prevalence and antimicrobial resistance profiles of Salmonella species and Escherichia coli isolates from poultry feeds in Ruiru Sub-County, Kenya
Source: BMC Res Notes. 2021 Feb 2;14:41. doi: 10.1186/s13104-021-05456-4 (PMC7852182; doi:10.1186/s13104-021-05456-4)
Supplement: Supplementary file 6 — Additional file 6: Table S2. Distribution of resistance genes. [file 13104_2021_5456_MOESM6_ESM.docx]

**Table S2:** Distribution of antimicrobial resistance genes among 34 isolates

|  |  | **Distribution of antimicrobial resistance genes among 34 isolates** | | | | | | |
| --- | --- | --- | --- | --- | --- | --- | --- | --- |
| **Antibiotic class** | | **Betalactam** | | **Sulphonamide** | | **Aminoglycoside** | |  |
| Gene present | | *TEM* | *SHV* | *Dfr* |  | *strB* |  |  |
| Total |  | 8(24%) | 4 (12%) | 7 (21%) |  | 3 (8%) |  |  |
